# Prevalence and predictors of parental distress at the communication of positivity at newborn screening for metabolic diseases: an Italian longitudinal study

**DOI:** 10.1136/bmjpo-2024-003103

**Published:** 2024-12-12

**Authors:** Marco Bani, Selena Russo, Serena Gasperini, Viola Crescitelli, Francesca Menni, Francesca Furlan, Francesco Tagliaferri, Graziella Cefalo, Sabrina Paci, Giuseppe Banderali, Paola Marchisio, Andrea Biondi, Maria Grazia Strepparava

**Affiliations:** 1 School of Medicine and Surgery, University of Milan-Bicocca, Milano, Italy; 2 Department of Pediatrics, Fondazione IRCCS San Gerardo dei Tintori, Monza, Italy; 3 Fondazione IRCCS Ca' Granda Ospedale Maggiore Policlinico, Clinical Metabolic Reference Center, Milan, Italy; 4 Pediatric Unit, Department of the Woman and Child, ASST Santi Paolo e Carlo, Milano, Italy; 5 Department of Pediatrics, IRCCS San Gerardo dei Tintori Foundation Hospital, Monza, Italy; 6 Clinical Psychology Unit, Fondazione IRCCS San Gerardo dei Tintori, Monza, Italy

**Keywords:** Psychology, Caregivers, COVID-19, Neonatology, Health services research

## Abstract

**Background:**

Receiving communication of positivity for metabolic diseases at Expanded Newborn Screening can be extremely stressful for parents, both in case of false positive and true positive cases. However, little is known about the predictors of distress and differential impact on mothers and fathers.

**Methods:**

In this longitudinal study, 169 fathers and 171 mothers referred to one of the Italian metabolic centres for communication of positivity completed a survey including General Health Questionnaire-12, Emotion Thermometers (measuring stress, anxiety, depression, anger and need for help), Impact of Event Scale–Revised, Multidimensional Scale of Perceived Social Support and Emotion Regulation Questionnaire. Perceived severity and control of the children’s health were also assessed. The survey was completed in person after the first session at metabolic centres and online after 1, 3 and 6 months.

**Results:**

Nearly 80% of parents reported a clinical level of distress and anxiety after the communication of positivity, one-third of them reported post-traumatic symptoms and more than half of parents reported a need for help. After 6 months, there are still more than 30% of parents with a clinical level of distress and anxiety, 6% with post-traumatic symptoms and more than 20% who continue to express a need for help. No gender difference was reported and no differences emerged between pre-COVID-19 and post-COVID-19 periods for parental distress and post-traumatic symptoms.

Social support, perceived severity and control of the child’s health—but not gender or previous parental experience—predicted the post-traumatic symptoms at baseline while at 6 months the only significant predictor was perceived severity.

**Conclusion:**

Adequate psychological support should be provided from the initial communication for both parents and for true positive, false positive and variants of uncertain significance/heterozygous carrier cases.

**WHAT IS ALREADY KNOWN ON THIS TOPIC**

- Communication of positivity for metabolic diseases at Expanded Newborn Screening can be stressful for parents.

**WHAT THIS STUDY ADDS**

- 80% of parents reported a clinical level of distress and anxiety at baseline, and 30% still after 6 months.
- Both parents reported similar levels of distress and post-traumatic symptoms.
- Parental distress did not differ among true positive, false positive and variants of uncertain significance/heterozygous carrier cases.

**HOW THIS STUDY MIGHT AFFECT RESEARCH, PRACTICE OR POLICY**

- Parental distress should be assessed and monitored since the initial communication.
- Psychological support should be provided to parents according to their level of distress since the initial communication.
- The perceived severity and social support should be monitored to identify parents at higher risk of developing psychological distress.

## Introduction

 In 2022, 393 333 children were born in Italy, and the parents of 279 of these children received a confirmation of a diagnosis for one of the metabolic diseases included in the Expanded Newborn Screening (ENBS). However, 913 parents were recalled for an out-of-range result of newborn bloodspots, and the confirmation process requires different steps and time to be completed.

Since 2016, in Italy, ENBS has been freely available to all newborns for the screening of 49 metabolic diseases.^1^ This programme is an essential public health programme aiming at providing early diagnosis (and, if available, treatment) of genetic diseases to prevent the long-term consequences of inherited metabolic disorders.^2^ One side effect of the programme is the high number of families receiving initial positive results that, after a second round of testing, can be confirmed, classified as false positives, healthy carriers or variants of uncertain significance (VUS). Consequently, the quality and monitoring of the communication of these positive results are extremely important. If not handled adequately, this communication can have a detrimental effect on the well-being of parents and their relationship with the newborn.^3^,^6^

The communication of positive results is a process that can yield various diagnostic outcomes. These include false positive results, clear diagnostic confirmation and intermediate situations that may require genetic screening for the family, temporary monitoring of the child’s clinical condition or concluding the ENBS process.^7^ In all these cases, the psychological impact of receiving a positive result adds an additional layer of distress for families. They must cope, often for the first time, with the psychological, relational and health-related responsibilities required by their new parental role.

Previous studies have shown that mothers can report anxiety, distress and depression following the communication of a positive result. This negative impact is observed not only in confirmed positive cases but also in false positives.^8^,^10^ Additionally, a false positive result casts children at a higher risk of being hospitalised.^11 12^ These figures highlight that even a potential and unconfirmed threat to the newborns’ health is perceived as real, negatively impacting the resources parents need to care for their children, especially in the first months of life.^6^

However, some considerations needed to be made to better analyse these findings and the emerging picture. First, many studies focused on specific clinical conditions, such as cystic fibrosis, Pompe disease and Krabbe disease^13^,^15^ and reported retrospective data on parental distress and its impact on family functioning. Furthermore, most of these studies used qualitative methodologies,^9^ which are useful for identifying relevant psychosocial areas and their in-depth analysis, but not for estimating the prevalence of parental distress and enabling comparison with other studies. Studies employing cross-sectional or longitudinal designs often involved small samples, collected data retrospectively, sometimes years after the initial communication of positivity^16^,^18^ or used non-validated tools to quantify distress.^19^ Consequently, the results are somewhat inconsistent.^20^ Finally, almost all studies assessed the psychological impact of positive results among mothers, with few including the perspective of fathers. When fathers are included, they are often under-represented, making comparisons difficult.^21^ Understanding and mapping the availability of all family resources and concerns is crucial for planning interventions to prevent or mitigate distress.

When looking at the identification of psychosocial predictors of parental distress related to the communication of ENBS positive results to design tailored interventions to support parental well-being and shield the parent–children relationship, only one study has been found.^22^ It focused on a later stage of positivity (the mean age of children was 8.5 years) and found that child adaptive functioning, parental satisfaction with support and the difficulties parents experienced in meeting their child’s healthcare needs were predictors of parental distress. These existing data are only partial. More data are needed to thoroughly address the issues arising from the communication of positive ENBS results and to develop and implement successful interventions.

Considering the fragmented data and the incomplete picture of the psychological impact of communication of ENBS positive results, the present longitudinal study aims to contribute to the literature gap by:

- Quantifying the psychological impact of positive ENBS results for metabolic diseases at the initial communication of test results and after 6 months.
- Comparing parental distress levels between true positive, false positive and VUS cases/heterozygous carriers and exploring potential gender differences (mothers vs fathers) over time.
- Exploring whether the COVID-19 pandemic worsened the impact of communication of positive results.
- Identifying the psychological predictors of parental distress and assessing potential gender differences.

## Methods

### Study design, participants and procedure

340 parents who received communication of positive results for metabolic diseases through ENBS in Lombardy between 2019 and 2022 were referred to one of the three metabolic clinical centres (MCC) available in Lombardy Region and accepted to participate in the study.

After the initial communication with a paediatrician specialised in metabolic diseases, during which parents were informed about the positive result and its clinical implications (and, when relevant, referred to second-level testing), parents were invited to complete a ‘paper and pencil’ questionnaire after signing informed consent (T0). When both were present, mothers and fathers completed the questionnaire independently.

At 1 (T1), 3 (T2) and 6 months (T3), parents were contacted by the main researcher via email or phone text and invited to complete a short online questionnaire (detailed in the ‘Measure’ section), with mothers and fathers receiving separate links. A reminder was sent 1 week after each follow-up request.

### Patient and public involvement

No patients are involved.

### Measures

The baseline study battery included sociodemographic information (gender, age, nationality, number of children, marital status and education) and the following validated questionnaires:

- The Impact of Event Scale-Revised (IES-R)^23^: This 22-item self-report tool measures distress caused by traumatic events over the past 15 days. Responses are given on a 5-point scale ranging from ‘not at all’ to ‘extremely’. The IES-R provides a total score (0–88) and three subscales assessing intrusion, avoidance and hyperarousal symptoms. A score of 33 or higher suggests a possible diagnosis of post-traumatic stress disorder (PTSD), while a score of 24 or higher may indicate partial PTSD. In our sample, the IES-R showed good reliability (Cronbach’s alphas 0.992, 0.963, 0.956 and 0.948 at T0, T1, T2 and T3, respectively).
- The General Health Questionnaire 12 (GHQ-12)^24^: The 12-question self-report tool assesses the severity of mental health problems over the past 2 weeks. Responses are rated on a 4-point scale ranging from ‘always’ to ‘never,’ with higher scores indicating worse health. Total scores range from 0 to 36 with scores between 14 and 19 indicating evidence of distress and scores equal to or greater than 20 suggesting severe psychological distress. In our sample, the GHQ-12 showed good reliability (Cronbach’s alphas 0.533, 0.713, 0.809 and 0.700 at T0, T1, T2 and T3, respectively).
- The Emotion Regulation Questionnaire (ERQ)^25^: A 10-item self-report 2-subscale tool measuring an individual’s tendency to use cognitive reappraisal and emotion suppression to deal with emotional arousal. Responses are rated on a 7-point scale ranging from ‘strongly disagree’ to ‘strongly agree,’ with higher scores indicating greater use of the strategy. In our sample, the two subscales showed good reliability (reappraisal subscale Cronbach’s alpha=0.810; suppression subscale Cronbach’s alpha=0.778).
- The Emotion Thermometers (ET)^26^: ETs consist of four columns (distress, anxiety, depression and anger) that describe the amount of emotional upset experienced in the past week, along with a column for indicating the level of help needed. Responses are rated on a 10-point Likert scale ranging from ‘none’ (0) to ‘extreme’ (10), except for the help thermometer which ranges from ‘can manage by myself’ (0) to ‘desperately’ (10) with a cut-off greater than or equal to 4. In our sample, the ET showed good reliability (Cronbach’s alphas 0.745, 0.914, 0.879, and 0.915 at T0, T1, T2 and T3, respectively).
- The Multidimensional Scale of Perceived Social Support (MSPSS)^27^ is a 12-question self-report tool that measures an individual’s perception of support from three sources: family, friends and a significant other. Responses are rated on a 5-point scale ranging from ‘strongly disagree’ to ‘strongly agree,’ with higher scores indicating greater perceived social support. In our sample, the MSPSS total score showed good reliability (Cronbach’s alpha=0.906).
- Two additional ad hoc questions were included to measure the perceived severity of the children’s condition and the perceived control over their health. Responses were rated on a 7-point Likert scale ranging from ‘0=not at all’ to ‘7=extremely.’

Follow-up assessments at 1 (T1), 3 (T2) and 6 (T3) months included only the IES-R, GHQ-12, ET and the two ad hoc questions on perceived severity and control. The baseline survey took approximately 20 min, while subsequent evaluations took around 10 min.

### Statistical analysis

Analyses included estimations of means, SD and frequency distribution of the investigated variables. We contrasted participant groups using χ2 test, z-score test and unpaired t-test or Mann-Whitney U-test.

One-way ANOVAs (Analysis of Variance) and Kruskal-Wallis H Tests explored if there were significant differences in the study measures (IES-R, GHQ-12, ERQ, MSPSS, perception of disease severity, perception of control and ET scores) among parents attending the three metabolic centres considered.

McNemar’s tests were employed to longitudinally assess changes over time in the frequency of participants meeting clinical criteria for PTSD (IES-R) and severe distress (GHQ-12). A set of two-way mixed ANOVAs examined the statistical significance of changes in study variables at 6 months while controlling for gender. Data were checked for repeated measures ANOVA assumption violations; namely, the presence of extreme outliers together with normality assumption were inspected for both analyses; the ANOVA assumption of sphericity was also tested through Mauchly’s test.

A series of two-way ANOVA was performed to explore whether the COVID-19 outbreak impacted the perception of severity and control, IES-R scores and levels of distress as indicated by the GHQ-12 while controlling for gender at T0.

A set of multiple linear regression was performed with the IES-R total score as the dependent variable at the different assessment points. Model selection included major demographic factors, variables theoretically associated with the dependent variable and those variables found to have a significant relationship in bivariate analysis.

Given the small sample size of the subgroups considered, we opted for a significance level of p<0.01 to reduce the likelihood of type I errors.

All analyses were performed using SPSS V.28.

## Results

A total of 340 parents accepted to participate in the study (171 mothers and 169 fathers) and completed the baseline assessment (T0). The sample’s demographics are reported in table 1. For the follow-up assessments, 80 (23.5%) participants completed the survey at T1 (1 month), 100 (29.4%) at T2 (3 months) and 97 (28.5%) at T3 (6 months).

**Table 1:** Sample characteristics

Parents n=340	
Age in years (mean±standard deviationSD)	34.74 (±5.5)
Gender (n=339)	
Female	171 (50.3%)
Male	168 (49.7%)
Educational Level (n=269)	
Year 5	40 (14.9%)
Year 12	139 (51.7%)
Bachelor’s degree or higher	90 (33.5%)
Parental experience (n=269)	
First child	147 (54.6%)
Second+child	122 (45.4%)
Nationality (n=290)	
Italian	261 (90.0%)
Other	29 (10.0%)
Children n=316	
Diagnosis type	
Beta-oxidation cycle disorders	179 (56.6%)
Organic aciduriaSs	33 (10.4%)
Aminoacidopathies	22 (7.0%)
Biotinidase deficiency	30 (9.5%)
Galactosaemia	20 (6.3%)
Phenylketonuria	32 (10.1%)
Test results	
True positive	82 (25.9%)
False positive	150 (47.5%)
VUS cases/Hheterozygous carrier	84 (26.6%)

No difference has been found between parents attending the three metabolic centres for MSPSS, ERQ, GHQ-12, IES-R, ET scores, perception of severity and control at any assessment points.

### The psychological impact of positive results and changes over time

When considering the initial communication of positive test results (T0) (see online supplemental table 1), nearly 80% of parents reported levels of distress and anxiety at the emotional thermometers above the cut-off values, with mothers reporting statistically significantly higher levels compared with fathers. Mothers also reported higher levels of depression and need for help, although nearly half of fathers reported the same need. Focusing on post-traumatic symptoms after the initial communication of positive results, more than 30% of parents reported an IES-R total score above the clinical threshold for post-traumatic disorder, with no difference between mothers and fathers.

No other gender differences emerge at baseline.

To explore changes over time in the perception of severity and control, a set of two-way mixed ANOVAs was conducted to determine whether there was a statistically significant difference in the study variables over 6 months (T0 and T3 were considered) while controlling for gender interactions. A statistically significant higher perception of the severity of positive results at T0 when compared with T3 has been found (F(1, 87)=41.099, p<0.001, partial η^2^=0.321) but no main effect of gender (F(1, 87)=1.083, p=0.301, partial η^2^=0.012) nor interaction effect (F(1, 87)=1.106, p=0.296, partial η^2^=0.013) emerged.

No statistically significant main effect of time (F(1, 88)=3.784, p=0.055, partial η^2^=0.041) and gender (F(1, 88)=0.06, p=0.808, partial η^2^=0.001) nor interaction effect (F(1, 88)=0.007, p=0.931, partial η^2^=0.000) have been found on parents’ perceived control.

Furthermore, two McNemar’s tests were run to determine if the proportion of parents scoring equal to or higher than the clinical cut-off of the IES-R and GHQ-12 were different at T3 compared with T0.

The proportion of parents reporting IES-R scores above the clinical cut-off had decreased from 32.5% at T0 to 6.2% at T3. The decrease was statistically significant, χ^2^ (1)=15.750, p<0.001.

As for the GHQ-12, the number of parents experiencing mild or severe distress had decreased from 43% at T0 to 28.8% at the 6-month follow-up. The decrease was statistically significant, χ2 (1)=8.828, p=0.003.

**Figure 1:**
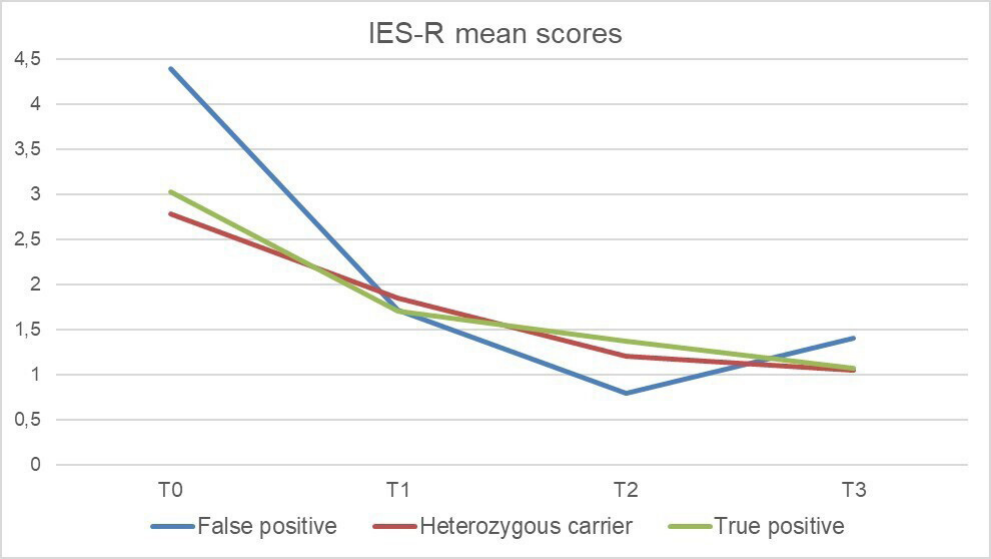
IES-R mean scores by test result categories across assessment point. IES-R, Impact of Event Scale-Revised.

Two two-way mixed ANOVAs explored changes between T0 and T3 in IES-R and GHQ-12 scores across the different possible positive results, namely, true positive, false positive and VUS cases/heterozygous carrier. A statistically significant main effect of time (higher IES-R at T0 when compared with T3) has been found (F(1, 86)=79.310, p<0.001, partial η^2^=0.480), while no main effect of the test result category emerged (F(2, 86)=3.207, p=0.045, partial η^2^=0.069). No interaction effect of time and test result category (F(1, 86)=1.957, p=0.148, partial η^2^=0.044) emerged (figure 1). When considering GHQ-12 scores, a statistically significant main effect of time has been found at T0 (F(1, 84)=7.453, p=0.008, partial η^2^=0.081). No main significant effect of test result category (F(2, 84)=1.340, p=0.267, partial η^2^=0.031) or interaction effect of time and test result category (F(1, 84)=0.444, p=0.643, partial η^2^=0.010) emerged.

### Impact of the pandemic on parental distress

The study enrolment started in 2018, and with the outbreak of the COVID-19 pandemic in Italy in March 2019, we had the opportunity to verify if the distress caused by the pandemic was an additional distressing factor to the initial communication of positive test results. A series of two-way ANOVA was performed to verify the impact of the COVID-19 outbreak on the perception of severity and control, and IES-R scores while controlling for gender at T0. No statistically significant interaction was found between pre-COVID-19 and post-COVID-19 outbreak and gender for perceived control score (F(1, 268)=0.119, p=0.731, partial η^2^=0) nor for perceived severity score (F(1, 268)=0.399, p=0.528, partial η^2^=0.001). Similarly, for IES-R, there was no statistically significant difference in mean score between males and females who received communication of positive results during the pandemic, F(1, 276)=4.106, p=0.044, partial η^2^=0.015.

### Psychological predictors of parental distress at different assessment points

A set of multiple linear regression was performed with IES-R total score as dependent variable and gender, previous parental experience, ERQ Suppression, ERQ Reappraisal, MSPSS, perceived severity and control as independent variables at the four assessment points.

At T0, the model statistically significantly predicted IES-R scores (F(7, 241)=8.831, p<0.001, adjust R*^2^*=0.181), in particular, higher perceived severity, lower perceived control and lower social support predicted the impact of the initial communication of positive test results. No effect emerged for gender nor for previous parental experience. At T1, the model resulted statistically significantly (F(7, 60)=3.582, p=0.003, adjust R*^2^*=0.212), with only perceived severity predicting IES-R scores. At T2 and at T3, the multiple regression models did not statistically significantly predict IES-R scores (F(7, 79)=2.454, p=0.025, adjust R^2^=0.106 at T2; F(7, 79)=2.141, p=0.049, adjust R^2^=0.085 at T3).

Regression coefficients and SEs are reported in table 2.

**Table 2:** Multiple regression results for IES-R total score at different assessment points

T0	B	95% CI for *B*		*SE B*	β	*R^2^*	*Δ^2^*
		*LL*	*UL*				
ModelConstant	5.52***	2929	8119	1317		0.204	0.181***
Gender	−0.421	−0.945	−0.104	−0.266	−0.096		
Parental experience	−0.164	−0.667	−0.339	−0.256	−0.037		
ERQ-Suppression	−0.155	−0.044	−0.354	−0.101	−0.097		
ERQ-Reappraisal	−0.123	−0.113	−0.359	−0.120	−0.062		
MSPSS	−0.546***	−0.871	−0.220	−0.165	−0.207***		
Perception of severity	−0.459***	−0.295	−0.622	−0.083	−0.328***		
Perception of control	−0.165^*^	−0.322	−0.009	−0.079	−0.123^*^		

T1	B	95% CI for *B*		*SE B*	β	*R^2^*	*Δ^2^*
		*LL*	*UL*				
ModelConstant	−1,318	−6,975	4339	2828		0.295	0.212**
Gender	−0.002	−1,092	1088	0.545	0.000		
Parental experience	−0.257	−1,339	0.825	0.541	−0.055		
ERQ-Suppression	0.208	0.221	0.637	0.214	0.124		
ERQ-Reappraisal	0.054	−0.437	0.544	0.245	0.027		
MSPSS	0.153	−0.621	0.926	0.387	0.052		
Perception of severity	0.752***	0.380	1125	0.186	0.474***		
Perception of control	−0.165	−0.446	0.115	0.140	−0.133		

T2	B	95% CI for *B*		*SE B*	β	*R^2^*	*Δ^2^*
		*LL*	*UL*				
ModelConstant	5148	0.404	9892	2383		0.179	0.106*
Gender	−0.682	−1,501	0.138	0.412	−0.172		
Parental experience	0.073	−0.725	0.870	0.401	0.019		
ERQ-Suppression	0.111	−0.205	0.428	0.159	0.080		
ERQ-Reappraisal	0.065	−0.310	0.440	0.188	0.038		
MSPSS	−0.612	−1,212	−0.012	0.302	−0.243		
Perception of severity	0.304	−0.010	0.618	0.158	0.218		
Perception of control	−0.041	−0.263	0.181	0.112	−0.042		

T3	B	95% CI for *B*		*SE B*	β	*R^2^*	*Δ^2^*
		*LL*	*UL*				
ModelConstant	3156	−0.769	7081	1972		0.159	0.085*
Gender	0.107	−0.556	0.770	0.333	0.034		
Parental experience	−0.143	−0.813	0.527	0.337	−0.046		
ERQ-Suppression	−0.025	−0.294	0.244	0.135	−0.021		
ERQ-Reappraisal	−0.035	−0.332	0.262	0.149	−0.026		
MSPSS	−0.281	−0.797	0.235	0.259	−0.128		
Perception of severity	0.327^*^	0.072	0.583	0.128	0.275		
Perception of control	−0.160	−0.339	0.018	0.090	−0.195		

Model=‘Enter’ method in SPSS.

* p<0.05, **p<0.01, ***p<0.001.

Bunstandardised regression coefficientERQEmotion Regulation QuestionnaireLLlower limitMSPSSMultidimensional Scale of Perceived Social SupportR2coefficient of determination∆R2adjusted coefficient of determinationSE BSE of the coefficientULupper limitβstandardised coefficient

## Discussion

This longitudinal study provided information on parents’ impact on the communication of positive ENBS results for metabolic diseases in Italy. The study aimed to quantify the psychological impact of positivity and its development in the first 6 months after communication, compare the level of distress of mothers and fathers, verify the impact of the COVID-19 pandemic on the psychological distress of positive test results and identify the psychological predictors of parental distress.

The results highlighted that more than 80% of parents reported a clinical level of distress and anxiety at baseline, and after 6 months, more than 30% still reported distress and anxiety. For one-third of parents, the impact of the communication of positivity is so deep as to cause post-traumatic symptoms that are still present after 6 months for 6% of them and require proper treatment to avoid consequences on the familiar well-being and the development of the parent–children relationship.^6^

Notably, no differences emerged between true positive, false positive and VUS cases/heterozygous carriers in terms of psychological distress and post-traumatic symptoms, highlighting that the impact of communication of positivity is disruptive independently by the confirmation of positivity.

These data echo a recent study on a small sample of Italian parents that found an 80% prevalence of anxiety at baseline but did not provide information on the development over time.^28^

Contrary to our expectations, a previous parental experience does not represent a protective factor and does not reduce the psychological impact of communication of positivity for parents.

The present study specifically tested the protective role of previous parental experience. Results showed the disrupting impact of communication of positivity for metabolic diseases, highlighting the need for adequate psychosocial support for both parents in the first months after the communication independently by having or no other children. Our results differ from those reported in a German study, including parents receiving communication of positivity of cystic fibrosis, where authors reported that parents with a firstborn and higher education were more worried.^13^

Another aim of the study was to compare the level of distress of mothers and fathers because almost all the previous studies were focused solely on mothers.

Our results showed for the first time that both mothers and fathers reported a similar level of distress and post-traumatic symptoms after communication of positivity, and the similarity was maintained in the following 6 months. Furthermore, mothers reported a higher level of anxiety and depression than fathers at baseline, as reported by a recent Italian study.^28^ However, in the 1, 3, and 6 months follow-up, these differences disappeared, and parents reported a similar level of anxiety and depression.

These results underscore the importance of incorporating routine assessments of parental distress into the communication process. Such assessments can help identify parents experiencing clinical levels of distress and guide the provision of tailored interventions such as psychoeducation, trauma-focused interventions and emotion regulation strategies, based on their specific needs. Furthermore, it is crucial to ensure the involvement of both parents throughout every stage of the communication process.^29^ This approach should also consider the potential of telehealth interventions to enhance accessibility and support for families during this critical period while leveraging their cost-effectiveness compared with in-person services.

We were also interested in verifying if the COVID-19 outbreak represented a supplementary distressing factor for parents receiving communication of positivity. While parents had to deal with the social, emotional and psychological requests of childbirth, those receiving communication of positivity at ENBS experienced a threat to their child. After the pandemic outbreak, parents have the additional burden of the limitations and danger posed by the pandemic (eg, access to the clinical metabolic centres limited to one parent, mandatory use of facemasks and risk of infection for themselves and the newborn). Contrary to our expectations, results showed that the impact of positivity was so profound that the additional distress caused by the pandemic did not change the overall parental distress. This result mirrors those relative to the previous parental experience and, one more time, shows that the threat to the baby represented by communication of positivity is so disruptive that it requires timely, specialised and continuous psychological support from the beginning of the process. Such support can help families with good resources to cope functionally with the distress experienced in the first step of the communication process and to identify parents at risk for long-term distress.^29^

The final aim of the study was to identify the predicting variables of parental distress, and the only variables predicting the post-traumatic symptoms after the communication of positivity are the lack of social support, the perception of the severity of the clinical condition of the child, the perception of control on his/her health.

This result suggests that interventions should be implemented to improve social support and perceived control of the child’s health and reduce the perceived severity. In particular, while there is no way to improve the social support from family and friends, the possibility of contacting a psychological service at first and associations of families with similar experiences can be a way to improve the perceived social support. Furthermore, the perceived severity and control of the child’s health condition are strictly related to how parents receive the communication, and the opportunity to clarify doubts and uncertainties represents a key factor in modulating these factors. For this reason, communication training to give information about positivity is needed, as reported in recent studies.^3^ Further studies are needed to identify which aspects of the communication process most significantly impact perceived severity, as well as which parental factors (eg, personality traits and knowledge about ENBS) are associated with a heightened perception of severity. These data could inform the development of evidence-based recommendations for clinicians tasked with communicating positive screening results.

### Limitations

Some limitations must be considered. First, the high dropout rate at follow-up assessment limited the strengths and generalisability of results; while parents completed the baseline assessment in ‘paper and pencil’ format at MCC, the subsequent assessments were done online, and parents were contacted by email the principal investigator that they have never met. This lack of direct knowledge can explain the low compliance to the following assessments, as well as the classification as false positive cases for many parents. Another reason for the high dropout rate was delayed communication of new enrolments; after the COVID-19 outbreak, MCCs had to manage many organisational changes in the clinical activities and reduce the timeliness of new enrolment (sometimes up to 3–6 months).

Another limitation relies on the 6 month follow-up, which captures the immediate impact of the communication of positivity on parents but fails to detect the long-term impact on parents and their interaction with children. A longitudinal study is needed to overcome this limitation, as is the inclusion of a control group of parents with a negative result at ENBS. A recent longitudinal German study^19^ on positive cases showed that parental psychosocial burden decreased by age but was higher in the first 3 years of children’s life and remained higher for patient groups with necessary dietary treatment.

Furthermore, we selected participants from a sample of parents whose children had to undergo a second test and subsequently attend an in-person visit at MCC. We did not include parents who received the first test result by phone but did not need to attend the in-person visit at the MCC as the children tested negative on the second test, so we do not know if the impact on parents receiving only the first phone communication is similar to that of parents who came to the MCCs.

## Conclusions

Parents receiving communication of positivity should always receive psychological support in the first step of the communication process. Regular screening for psychological distress could help identify parents most in need of help and address resources toward more frail parents (those with limited social support and a higher perception of the severity of their children’s condition).

## supplementary material

10.1136/bmjpo-2024-003103 online supplemental file 1

## Data Availability

Data are available on reasonable request.
