# Supplementary material for: Prevalence and predictors of parental distress at the communication of positivity at newborn screening for metabolic diseases: an Italian longitudinal study
Source: BMJ Paediatr Open. 2024 Dec 12;8(1):e003103. doi: 10.1136/bmjpo-2024-003103 (PMC11667425; doi:10.1136/bmjpo-2024-003103)
Supplement: online supplemental file 1 [file bmjpo-8-1-s001.pdf]

	T0			T1			T2			T3		
	Mothers N=171	Fathers N=169	TOT N=340	Mothers N=47	Fathers N=33	TOT N=80	Mothers N=62	Fathers N=38	TOT N=100	Mothers N=59	Fathers N=38	TOT N=97
GHQ-12	14.67 ±3.8	14.93 ±3.6	14.79 ±3.7	14.78 ±4.1	14.36 ±4.2	14.61 ±4.1	15.16 ±4.5	12.76 ±3.4 *	14.25 ±4.3	13.68 ±3.8	13.42 ±2.6	13.57 ±3.4
Distress %	40 (26.5%)	45 (35.7%)	85 (30.7%)	13 (27.7%)	7 (21.2%)	20 (25%)	19 (30.6%)	5 (13.2%)*	24 (24%)	10 (16.9%)	11 (28.9%)	21 (21.6%)
Severe Distress %	21 (13.9%)	13 (10,3%)	34 (12.3%)	7 (14.9%)	4 (12.1%)	11 (13.8%)	9 (14.5%)	2 (5.3%)*	11 (11%)	6 (10.2%)	1 (2.6%)	7 (7.2%)
IES-R	3.58 ±2.2	3.14 ±2.2 *	3.38 ±2.2	1.86 ±2.1	1.79 ±2.2	1.83 ±2.1	1.95 ±2.0	1.24 ±1.7 *	1.68 ±1.9	1.09 ±1.5	1.15 ±1.6	1.11 ±1.5
IES-R ≥ clinical threshold %	51 (33.6%)	40 (31.3%)	91 (32.5%)	4 (8.5%)	5 (15.2%)	9 (11.3%)	8 (12.9%)	2 (5.3%)	10 (10%)	3 (5.1%)	3 (7.9%)	6 (6.2%)
Perceived Control	4.59 ±1.7	4.56 ±1.6	4.58 ±1.7	4.16 ±1.8	4.07 ±1.8	4.13 ±1.8	4.22 ±1.8	4.74 ±2.2	4.42 ±1.9	5.09 ±1.8	5.21 ±2.0	5.14 ±1.3
Perceived Severity	3.53 ±1.6	3.09 ±1.6 *	3.33 ±1.6	2.91 ±1.3	2.83 ±1.5	2.88 ±1.4	2.57 ±1.3	2.23 ±1.6	2.44 ±1.4	2.09 ±1.4	1.95 ±1.2	2.03 ±1.9
Emotional Thermometers												
Stress	6.39 ±2.5	5.68 ±2.9 *	6.06 ±2.7	3.60 ±2.3	4.06 ±2.6	3.79 ±2.4	3.39 ±2.9	3.44 ±2.5	3.41 ±2.8	2.80 ±2.5	2.71 ±2.5	2.76 ±2.5
Stress >4 %	116 (83.5%)	87 (74.4%)*	203 (79.3%)	25 (53.2%)	15 (45.4%)	40 (50%)	25 (40.3%)	19 (50%)	44 (44%)	20 (33.9%)	13 (34.2%)	33 (34%)
Anxiety	6.56 ±2.8	5.32 ±2.9 **	6.00 ±2.9	3.06 ±2.5	3.09 ±2.5	3.08 ±2.5	3.11 ±3.1	2.82 ±2.3	3.00 ±2.8	2.63 ±2.4	2.26 ±2.2	2.48 ±2.3
Anxiety > 4 %	118 (85.5%)	79 (68.7%) **	197 (77.6%)	19 (40.4%)	9 (27.3%)	28 (35%)	23 (37.1%)	18 (47.4%)	41 (41%)	21 (35.6%)	10 (26.3%)	31 (32%)
Depression	2.77 ±2.7	1.89 ±2.6 *	2.36 ±2.7	1.47 ±2.2	1.61 ±2.2	1.53 ±2.2	1.44 ±2.1	0.71 ±1.3	1.16 ±1.9	1.39 ±2.1	0.50 ±1.1 *	1.04 ±1.8
Depression >4 %	54 (39.1%)	23 (19.7%) **	77 (30.2%)	8 (17%)	4 (12.1%)	12 (15%)	10 (16.1%)	3 (7.9%)	13 (13%)	8 (13.6%)	2 (5.3%)	10 (10.3%)

Anger	3.22 ±3.2	3.57 ±3.3	3.38 ±3.2	2.06 ±2.6	1.97 ±2.5	2.03 ±2.5	2.03 ±2.6	1.82 ±2.4	1.95 ±2.5	1.58 ±2.2	1.21 ±1.7	1.43 ±2.0
Anger >4 %	53 (38.4%)	46 (39.9%)	99 (38.8%)	9 (19.1%)	8 (24.2%)	17 (21.3%)	13 (21%)	8 (21%)	21 (21%)	11 (18.6%)	6 (15.8%)	17 (17.5%)
Need for help	4.35 ±2.8	3.63 ±3.02 *	4.02 ±2.9	3.13 ±2.4	2.45 ±2.4	2.85 ±2.4	2.86 ±2.5	1.63 ±2.0 *	2.39 ±2.4	2.19 ±2.4	1.76 ±1.9	2.02 ±2.2
Need for help >4 %	85 (61.6%)	56 (48.7%)*	141 (55.7%)	16 (34%)	9 (27.3%)	25 (31.3%)	24 (38.7%)	9 (23.7%)	33 (33%)	15 (25.4%)	8 (21.1%)	23 (23.7%)

Supplementary table 1 - Frequencies and values of psychological distress (GHQ-12), post-traumatic symptoms (IES-R), emotion thermometers (ET), perceived severity, and perceived control scores by gender and over time. $^{**}p<.001$; $^{*}p<.05$
