# Supplementary material for: Prevalence and predictors of parental distress at the communication of positivity at newborn screening for metabolic diseases: an Italian longitudinal study
Source: BMJ Paediatr Open. 2024 Dec 12;8(1):e003103. doi: 10.1136/bmjpo-2024-003103 (PMC11667425; doi:10.1136/bmjpo-2024-003103)
Supplement: Supplementary file 2 [file bmjpo-2024-003103.reviewer_comments.pdf]

PEER REVIEW HISTORY

BMJ Paediatrics Open publishes all reviews undertaken for accepted manuscripts. Reviewers are asked to complete a checklist review form and are provided with free text boxes to elaborate on their assessment. These free text comments are reproduced below.

ARTICLE DETAILS

**TITLE (PROVISIONAL)**	Prevalence and predictors of parental distress at the communication of positivity at newborn screening for metabolic diseases: an Italian longitudinal study
**AUTHORS**	Bani, Marco; Russo, Selena; Gasperini, Serena; Crescitelli, Viola; Menni, Francesca; Furlan, Francesca; Tagliaferri, Francesco; Cefalo, Graziella; Paci, Sabrina; Banderali, Giuseppe; Marchisio, Paola; Biondi, Andrea; STREPPARAVA, MARIA GRAZIA

VERSION 1 - REVIEW

**REVIEWER NAME**	***Martina Huemer***
**REVIEWER AFFILIATION**	University Children's Hospital Zürich, Metabolic diseases
**REVIEWER CONFLICT OF INTEREST**	
**DATE REVIEW RETURNED**	18-Oct-2024

**GENERAL COMMENTS**	This is important work that focuses our attention on the psychological impact of NBS programmes and recalls. I would not consider a recall<br>Page 4, Conclusion: Is psychological support the same as social support? You are outlining that little social support predicts higher stress levels but not the lack of psychological support. Please consider and discuss - does your data support this conclusion? Would offering psychological support this be a realistic and applicable approach in the clinical setting?<br>Page 5, l 28: Not all with a positive screening result that are not confirmed or false, are carrying VUS. Please correct.<br>Page 7 L22: Why is COVID treated so special? It should be one of the predictors "communication of diagnosis during the pandemic"<br>Method, P8: My main criticism is that the way of contacting families, communicating about the recall, and the measures that were taken are not specified. It would have been most interesting to see whether a standardised way of contacting and communicating the families would have mad a difference. I would like to relate this to my comment above: providing psychological support for everyone may not be easy to achieve but the development of psychologically informed, standardised ways of communicating with parents in this vulnerable phase could make a real difference. You probably have more data on "who did what and how". Please add them to the manuscript and, if possible, analyse them along with the other predictors. Is there any relation between the invasiveness / quantity / number of tests (maybe also genetic testing involved early) required at recall and the parameters of interest?

REVIEWER NAME	***Camilla Pisoni***
**REVIEWER AFFILIATION**	Fondazione IRCCS Policlinico San Matteo
**REVIEWER CONFLICT OF INTEREST**	
**DATE REVIEW RETURNED**	07-Nov-2024

GENERAL COMMENTS	Dear authors, the work is very well structured and written. I have no revision to ask for. I can say that it brings important information about the mental state of parents receiving metabolic disease screenings. The publication also provides interesting insights into the care of these parents over time. Thank you for your work.

VERSION 1 – AUTHOR RESPONSE

Reviewer: 1

Prof. Martina Huemer, University Children's Hospital Zürich

Comments to the Author

This is important work that focuses our attention on the psychological impact of NBS programmes and recalls. I would not consider a recall

Page 4, Conclusion: Is psychological support the same as social support? You are outlining that little social support predicts higher stress levels but not the lack of psychological support. Please consider and discuss - does your data support this conclusion? Would offering psychological support this be a realistic and applicable approach in the clinical setting?

Response: Thank you for your insightful comment, which allowed us to clarify the distinct roles of psychological and social support in our study and the clinical implications of our findings. We agree that social and psychological needs should be distinguished. Our results specifically identify the lack of social support as a predictor of baseline distress. However, the elevated distress and post-traumatic symptoms reported by many parents indicate a clear need for psychological interventions. We have therefore revised our discussion to highlight the importance of routine screening for distress in all parents from the initial communication, as well as the need for psychological interventions tailored to the parents' needs and distress levels. We agree that long term psychological support for all the parents cannot be realistic; however, a single session after the first communication or the presence of a psychologist during the communication is feasible and should be considered.

We did not include psychological support as a predictor variable in this study as, at the time of data collection, it was offered only at the clinicians' request and to a limited subset of parents.

Page 5, l 28: Not all with a positive screening result that are not confirmed or false, are carrying VUS. Please correct.

Response: Thank you for this comment. We agree with the reviewer, and we have revised the sentence to accurately reflect all possible screening outcomes, including VUS cases.

Page 7 L22: Why is COVID treated so special? It should be one of the predictors "communication of diagnosis during the pandemic"

Response: Thank you for this valuable comment that allows us to better explain our rationale for examining the impact of COVID-19 pandemic, which was not one of the study’s original aim. As described in the Results section "Impact of the pandemic on parental distress," the COVID-19 outbreak provided an opportunity to explore whether an additional distressing event like the pandemic worsened the parental distress associated with receiving a positive screening result. Our results showed that parental distress was similar before and during the pandemic, suggesting that "the additional distress caused by the pandemic did not change the overall parental distress".

To our knowledge, no other studies on newborn screening have assessed the pre/post-COVID exposure, making this a unique aspect of our study. Initially, we chose not to include the pandemic period (pre/post) as a predictor in the regression analysis on distress, as this variable had already been extensively investigated through ANOVAs to assess its potential influence on distress, perceived severity, and perceived control. However, for clarity and completeness, the regression analysis was repeated to include the COVID pre/post variable, which confirmed that it is not a predictor of parental distress. This analysis is attached to this response letter but we preferred not include it in the manuscript to avoid a duplication of the results presented through ANOVA. However, we remain available to integrate it in the manuscript if requested.

Method, P8: My main criticism is that the way of contacting families, communicating about the recall, and the measures that were taken are not specified. It would have been most interesting to see whether a standardised way of contacting and communicating the families would have mad a difference. I would like to relate this to my comment above: providing psychological support for everyone may not be easy to achieve but the development of psychologically informed, standardised ways of communicating with parents in this vulnerable phase could make a real difference. You probably have more data on "who did what and how". Please add them to the manuscript and, if possible, analyse them along with the other predictors. Is there any relation between the invasiveness / quantity / number of tests (maybe also genetic testing involved early) required at recall and the parameters of interest?

Response: Thank you for these comments. The process of contacting families within the Italian ENBS is detailed in a previous publication (Bani M, Russo S, Raggi E, et al. Parents' experience of the communication process of positivity at newborn screening for metabolic diseases: A qualitative study. Child Care Health Dev. 2023;49(6):961-971. doi:10.1111/cch.13105) where we focus on the critical role of the initial phone call from the newborn screening centers. We do agree with the reviewer that the way of communicating positive screening results can significantly impact parental experience and distress. However, at this stage, we have no additional data on specific communication methods and strategies used in each case, and so we cannot add further details to the manuscript. We, nevertheless, are currently investigating this topic through a qualitative project using semi-structured interviews.

While there are no existing guidelines for standardized communication within newborn screening in Italy, all communication in our study was handled by the six clinicians across three metabolic centers. This consistency likely contributed to a coherent approach, as no significant differences in parental distress were observed across centers. To address the reviewer's suggestions, we have added a paragraph in the discussion highlighting the potential benefit of developing evidence-based, standardized, and psychologically informed communication methods as a key factor in supporting parents through this vulnerable phase.

Reviewer: 2

Dr. Camilla Pisoni, Fondazione IRCCS Policlinico San Matteo

Comments to the Author

Dear authors, the work is very well structured and written. I have no revision to ask for. I can say that it brings important information about the mental state of parents receiving metabolic disease screenings. The publication also provides interesting insights into the care of these parents over time. Thank you for your work.

Response: Thank you for this encouraging feedback. We are committed to contribute to the literature on the impact of neonatal screening and to the ongoing effort to support impacted families.
